# Affirmative action, financial support, and medical residency outcomes in Brazil: evidence from a national linked cohort study, 2018-2024

**DOI:** 10.1186/s12909-026-08644-7

**Published:** 2026-01-24

**Authors:** Paola Soledad Mosquera, Mário César Scheffer, Alicia Matijasevich, Ivan Wilson Hossni Dias, Giuliano Russo

**Affiliations:** 1 Preventative Medicine Department, University of São Paulo, Avenida Dr. Arnaldo, 455; 2º andar; sala 2214, São Paulo, Brazil; 2 Wolfson Institute of Population Health, Queen Mary University of London, 58 Turner Street, London, E1 2AB UK

**Keywords:** Medical education, Medical specialists, Affirmative action, Quotas, Medical scholarships, Brazil, Low- and middle-income countries (LMICs)

## Abstract

**Background:**

Medical education and workforces lack heterogeneity worldwide due to socioeconomic barriers, biased recruitment, and structural inequalities. In Brazil, affirmative action (AA) policies aim to address these disparities through quota-based admissions in public universities and financial aid in private institutions. While these measures may diversify the student pipeline, their influence on medical residency (MR) entry and specialty choice is less clear, with implications for those primary care specialties central to health system performance. This study assessed whether AA students in Brazil are less likely to enter MR programs and whether their specialty selection, particularly Family & Community Medicine, differs from peers.

**Methods:**

We conducted a retrospective cohort study using nationally linked data. Medical graduates were identified from the Higher Education Census (2018–2022) and followed for medical residency outcomes using National Medical Residency Commission records from 2019 to 2024. Exposures were: quota admission in public schools, and; financial support in private schools. Outcomes included entry into a nationally accredited MR program and, among those entering residency, selection of Family & Community Medicine. Logistic regression models estimated adjusted odds ratios (aORs) with 95% confidence intervals, controlling for demographic (sex, race, age, schooling background) and institutional factors (region, ENADE scores, school type, and age).

**Results:**

The study included 110,911 medical graduates. Among public school students, 28.1% entered via quotas; in private schools, 44.5% received financial support. Quota students were more likely to come from public secondary schools (95.3% vs. 20.1%) and were more racially diverse than peers, with 49.8% identifying as mixed-race and 12.1% as Black, versus 24.6% and 3.9% among non-quota peers. Residency entry was lower among quota students (52.8% vs. 66.7%; aOR: 0.73, 95% CI: 0.67–0.79), but higher among financial aid recipients (55.0% vs. 50.5%; aOR: 1.23, 95% CI: 1.19–1.27). Specialty distributions were broadly similar, though quota students were more likely to select Family & Community Medicine (15.3% vs. 9.7%; aOR: 1.28, 95% CI: 1.09–1.49). A smaller but significant effect was observed among financial aid recipients (8.2% vs. 6.9%; aOR: 1.09, 95% CI: 1.00–1.18).

**Conclusions:**

Quota policies increased diversity in Brazilian medical schools, especially by expanding the representation of Black and mixed-race students, but were associated with lower MR enrolment, possibly due to financial hardship. Financial aid in private schools facilitated residency entry. Both policies encouraged primary care specialty choice, particularly among quota students. These findings highlight the complementary roles of quotas and financial support in shaping workforce heterogeneity and specialty distribution in Brazil and other LMICs.

## Introduction and background

Worldwide, the medical workforce remains markedly homogeneous, with physicians from higher socioeconomic backgrounds and historically privileged racial groups disproportionately represented. This pattern reflects socioeconomic and academic barriers faced by underrepresented groups, biased recruitment policies, and structural inequalities within medical education and professional bodies [1]. Consequently, the medical workforce often does not reflect the diversity of the populations it serves, undermining trust with marginalized communities and reinforcing inequalities within health systems and broader society [2]. A growing body of literature indicates that lack of representativeness in the medical workforce is associated with lower trust in patient–provider relationships, the persistence of racism and implicit bias in care, and poorer adherence to treatment among marginalized populations [3, 4].

The lack of heterogeneity is particularly evident in the selection of medical specializations, with specialties like surgery seeing an over-representation of male, affluent, and white students [5], and other medical areas such as gynaecology and primary care been disproportionately chosen by female students, often from diverse racial and economic background [6].

The literature suggests that medical students’ specialty choices are influenced by personal factors (such as individual interests, values, and demographics), specialty characteristics (lifestyle, work-life balance, income, prestige), and clinical experiences [7]. From an economic stand point, known factors include a desire for controllable work hours, geographical location of practice, perceived job opportunities, financial rewards, and guidance from mentors and family [8]. All such factors have contributed to scarcity and imbalances in the distribution of specialists, complicating forecasts and training of the medical workforce, ultimately affecting the availability of specialist services in peripheral parts of health systems, particularly for low- and middle-income countries [9].

With a view to increasing access to medical training for individuals from population groups historically underrepresented in the profession, “affirmative action” (AA) policies have been implemented in high- and middle-income countries (LMIC) [10]. These policies often involved preferential or compensatory measures (such as admissions quotas, race/ethnicity-based selection, financial subsidies or scholarships), aimed at remedying past inequities, improving diversity in the medical workforce, and enhancing health equity in the society [11, 12]. AA measures have often been framed as ‘public policies’ when carried out through public institutions, or private sector ones when implemented through incentives for private schools to recruit and employ from disadvantaged populations [13].

Evidence from the economic literature broadly shows that race- and income-conscious affirmative-action policies increase enrolment and representation of under-represented racial/ethnic groups at selective institutions [14]. Studies on law-schools in the United States (US) [15] have reported evidence of a “mismatch” between quota students’ preparation and institutions’ high requirements. Other large empirical studies have found little or mixed evidence that AA causes net harm to beneficiaries [14, 16].

What impact AA policies have in the field of medical education is, however, contested. Studies from the US suggests AA measures help sustain a more racially and socioeconomically diverse pipeline of physicians, which, in observational studies has been associated with greater likelihood of practicing in underserved areas and improved patient-provider [17, 18]. On the other hand, evidence that affirmative-action admits perform substantially worse in medical school (a “mismatch” causing harm) is limited and mixed; a study from Brazil comparing social quota students to regular path students found no significant difference in attending medical residency programs, with social quota students often showing strong interest in rural practice and general practice [19].

In Brazil, social and racial quotas gradually expanded in the 2000es with the roll-out of the Student Financing Fund - FIES, 2001- providing low-interest loans for those enrolled in private institutions [20] and fees exemptions by the University for All Program (PROUNI, 2005), offering full and partial scholarships to low-income and non-white students from public high schools [21]. In 2012, Federal Law 12.711 mandated that federal universities reserve at least 50% of places for students from public schools, self-declared Black, mixed-race (Pardos), or Indigenous, those with family incomes up to 1.5 minimum wages [22]. Admission through quotas requires lower cutoff grades in the National High School Exam (ENEM), which some scholars arguing would affect students’ decisions to use quotas and contribute to stigma [23].

In 2022, 11.5% of the 5.1 million students enrolled participated in quota schemes, while 36.8% of the 3.2 million students in private institutions received some form of student financing. In medicine, however, a traditional course with some of the highest tuition fees in Brazil, these proportions were lower, with only 9% of students entering through quotas and 23.2% receiving financial aid [24].

Medical residency (MR) in Brazil represents the primary pathway for training medical specialists, although it is not mandatory for the practice of medicine as a generalist. In 2024, 47,718 physicians - approximately 8% of all practicing doctors - were enrolled in MR programs [24, 25]. These programs are publicly funded through government scholarships, filled via competitive selection processes, and characterized by training lasting two to five years, depending on the specialty. Those who do not pursue residency may practice as generalists without a specialty or follow an alternative specialization pathway by obtaining a specialty title from the Brazilian Medical Association (AMB), which requires proof of clinical experience and passing a certification exam.

The rapid expansion of private sector medical schools over the past decade has intensified competition for MR positions, as the number of training slots has not kept pace with the volume of new graduates [26]. Within this scenario, data from the 2025 Brazilian Medical Demographics Study indicated that the majority of physicians enrolled in MR in 2024 (62.8%) had graduated from private medical schools [25].

This study examines whether medical students admitted through affirmative action policies in Brazil are less likely to enter medical residency programs than their peers admitted via the regular track, and whether their specialty choices differ, particularly with regards to primary care practice (or Family & Community Medicine, as it is known in Brazil). Because affirmative action measures in Brazil have been implemented through public and private sector policies - quotas for underrepresented groups in public medical schools and financial subsidies for students in private medical schools - we analyse the effects of those policies separately.

By linking national data on medical school admission pathways to subsequent entry into medical residency and specialty choice, this study provides empirical evidence on the relative effectiveness of affirmative action policies across public and private medical education sectors in LMICs.

## Methods

We investigated the association between students’ participation in affirmative action (AA) programs during medical school - specifically admission through quota systems in public schools and receipt of financial aid in private schools - and subsequent entry into medical residency (MR) and choice of primary care specialty. Because socioeconomic profiles differ between students in public and private institutions [27], outcomes were analysed separately across four groups: [1] public school students admitted via quotas vs. regular admissions, and [2] private school students receiving financial aid vs. those without support.

### Data sources

We combined multiple datasets to construct sociodemographic and educational profiles of medical graduates and to examine AA program participation in relation to MR outcomes.

Data on medical graduates (students who have just completed medical school), from 2018 to 2022, was gathered from the Higher Education Census, collected annually by the National Institute of Educational Studies and Investigations Anísio Teixeira (INEP) [28]. We restricted analyses to 2018 onward, as beneficiaries of the 2012 Quota Law would be expected to graduate from 2018.

Information on resident physicians, from 2019 to 2024, was obtained from the National Medical Residency Commission of the Brazilian Ministry of Education (CNRM/MEC). Since MR programs begin in March, only graduates from 2018 could enroll starting in 2019.

Information on medical courses, including year of establishment and average ENADE 2023 score (National Exam of Students Performance) was obtained from the Ministry of Education and Culture database (e-MEC) [29]. Population estimates (2018–2022) for municipalities where medical schools are located were based on data from the Brazilian Institute of Geography and Statistics (IBGE) [30].

Access to INEP and CNRM databases was granted through the Protected Data Access Service (Sedap). Records were linked via encrypted individual identification numbers (CPF), in compliance with Brazil’s General Data Protection Law (Law No. 13,709/2018) [31].

### Exposures and outcomes

The exposures of interest were quota admission (public medical schools: yes/no), reserved for students who self-identify as Black, Indigenous or Mixed-race, from low-income family background, attended public secondary schools, or have disabilities [22], and financial aid receipt (private medical schools: yes/no), comprising refundable mechanisms (the FIES program, loans from medical school or external entities) and non-refundable mechanisms (full and partial PROUNI scholarships, as well as grants from the medical school or external entities). In the database, among the aid recipients, 10.7% were beneficiaries of the full PROUNI program. Students could meet multiple quota criteria or receive more than one type of aid; exposures were therefore analysed as binary variables.

The outcomes were entry into MR (yes/no) and, among those admitted, enrolment in Family & Community Medicine (yes/no). Entry into MR was defined as having at least one first-time admission to a medical residency program at any point during the follow-up period (2019–2024) after graduation. We analysed the first MR admission regardless of subsequent re-enrolments. At the time of data extraction, residents were classified as completed (47.7%), active (43.1%), withdrawn (8.7%), or other status (0.5%). Because only first admissions were included, all programs analysed corresponded to direct-entry specialties.

We adjusted analyses for socioeconomic characteristics of medical graduates: sex; self-reported race (IBGE categories: white, black, brown, indigenous, yellow), type of secondary school (public/private), and age at medical school entry (≤ 21 or > 21 years); educational characteristics of medical schools attended: medical school type (public/private); years of school operation (≤ 10, 11–20, ≥ 21); and ENADE 2023 score (high: 3–5 vs. low: 1–2); and geographic characteristics of training institutions: geographic region (North, Northeast, Southeast, South, Central-West), and municipal population size (> 300,000 vs. <300,000). Schools were classified as public if tuition-free; those charging tuition, with or without subsidies, were classified as private. Municipal population size refers to the municipality where the medical school attended by each student is located and was included as an individual contextual characteristic, reflecting differences in training settings, access to teaching hospitals and residency programs, and student profiles associated with the recent expansion of medical schools into smaller municipalities [25]. All characteristics were defined at the individual student level, although some variables describe attributes of the medical schools attended and their geographic context.

### Statistical analysis

We first described graduate characteristics (2018–2022) by AA participation, stratified by school type. Comparisons of entry into MR (2019–2024), choice of Family & Community Medicine, and distribution of major specialties were conducted using frequencies and proportions.

Logistic regression models estimated crude and adjusted odds ratios (aORs) with 95% confidence intervals (CIs) for associations between AA participation and outcomes, stratified by school type. All available covariates, considered conceptually relevant, were simultaneously included in multivariable models. Missing data for age at entry (0.06%), self-reported race (13.3%), secondary school type (0.8%), and ENADE score (3.3%) were handled via complete case analysis.

All analyses were performed using Stata 15.0 (StataCorp, College Station, TX, USA) and Microsoft Excel for Microsoft 365.

### Ethics approval and consent to participate

This study is in compliance with the Helsinki Declaration, and received approval from the Research Ethics Committee of the University of São Paulo Medical School (CAAE No. 71626323.8.0000.0068). Informed consent to participate was obtained from all the participants in the study.

## Results

Our database included 110,911 medical students that graduated between 2018 and 2022. With respect to socioeconomic characteristics (Table 1), the majority of students self-declared as White and had attended private secondary schools. Among students enrolled in public medical schools, those admitted through quota policies were considerably more diverse than their peers, with a lower proportion of Whites (35.6% vs. 66.8%) and higher proportions of mixed-race (49.8% vs. 24.6%) and Black students (12.1% vs. 3.9%). Quota students also disproportionately came from public secondary schools compared with non-quota students (95.3% vs. 20.1%). In private medical schools, recipients of financial support were also more diverse than their peers, although to a lesser extent, with fewer White students (71.2% vs. 78.9%) and higher proportions of mixed-race (22.9% vs. 16.8%) and Black students (3.1% vs. 1.7%). A higher proportion of scholarship recipients had attended public secondary schools (22.6% vs. 15.0%).

**Table 1:** Demographic and educational characteristics of medical graduates (2018–2022) in Brazil, by type of medical school (public or private) and participation in affirmative action programs

Characteristics	Medical graduates (total) *n* = 110,911	Public medical school graduates *n* = 34.844 (31.4%)		Private medical school graduates *n* = 76.067 (68.6%)	
		Admission through quotas *n* = 9,777 (28.1)	Admission through regular path *n* = 25,067 (71.9)	Recipients of financial aid *n* = 33,849 (44.5%)	Non-recipients of financial aid *n* = 42,218 (55.5)
Sex					
Female	65,337 (58.9)	4,820 (49.3)	12,806 (51.1)	21,298 (62.9)	26,413 (62.6)
Male	45,574 (41.1)	4,957 (50.7)	12,261 (48.9)	12,551 (37.1)	15,805 (37.4)
Age at start of graduation*					
≤ 21years	76,037 (68.6)	6,566 (67.2)	18,372 (73.3)	22,274 (65.8)	28,825 (68.3)
> 21 years	34,810 (31.4)	3,204 (32.8)	6,678 (26.7)	11,561 (34.2)	13.367 (31.7)
Self-reported race*					
White	67,092 (69.8)	3,290 (35.6)	13,997 (66.8)	20,407 (71.2)	29,398 (78.9)
Pardos (mixed-race)	22,616 (23.5)	4,610 (49.8)	5,162 (24.6)	6,564 (22.9)	6,280 (16.8)
Black	3,459 (3.6)	1,116 (12.1)	830 (3.9)	871 (3.1)	642 (1.7)
Yellow	2,510 (2.6)	107 (1.2)	858 (4.1)	660 (2.3)	885 (2.4)
Indigenous	465 (0.5)	123 (1.3)	119 (0.6)	152 (0.5)	71 (0.2)
Type of institution attended for secondary education*					
Public school	28,167 (25.6)	9,304 (95.3)	4,951 (20.1)	7,598 (22.6)	6,314 (15.0)
Private school	81,867 (74.4)	456 (4.7)	19,622 (79.9)	26,041 (77.4)	35,748 (85.0)
Region of the medical school					
North	7,968 (7.2)	605 (6.2)	3,102 (12.4)	1,279 (3.9)	2,982 (7.1)
Northeast	24,276 (21.9)	2,614 (26.7)	7,306 (29.1)	7,752 (22.9)	6,604 (15.6)
Southeast	53,621 (48.3)	3,687 (37.7)	8,826 (35.2)	17,517 (51.7)	23,591 (55.9)
South	16,285 (14.7)	1,779 (18.2)	3,713 (14.8)	4,959 (14.6)	5,834 (13.8)
Central-west	8,761 (7.9)	1,092 (11.2)	2,120 (8.5)	2,342 (6.9)	3,207 (7.6)
Medical school location by population size					
Areas with > 300.000 inhabitants	77,729 (70.1)	7,140 (73.0)	19,340 (77.1)	22,660 (66.9)	28,589 (67.7)
Areas with fewer than < 300.000 inhabitants	33,182 (29.9)	2,637 (27.0)	5,727 (22.9)	11,189 (33.1)	13,629 (32.3)
Length of operation of the medical school					
≥ 21 years	57,184 (51.5)	7,260 (74.3)	18,604 (74.2)	13,893 (41.0)	17,427 (41.3)
11 |-| 20 years	33,462 (30.2)	872 (8.9)	3,557 (14.2)	12,840 (38.0)	16,193 (38.3)
≤ 10 years	20,265 (18.3)	1,645 (16.8)	2,906 (11.6)	7,116 (21.0)	8.598 (20.4)
Medical school average ENADE 2023 score*					
Score 3–4-5 (higher)	87,308 (81.4)	9,162 (97.5)	21,501 (95.5)	25,242 (75.0)	31,403 (75.3)
Score 1–2 (lower)	19,957 (18.6)	230 (2.5)	1,006 (4.5)	8,423 (25.0)	10,298 (24.7)

* Variation in n is due to missing data: 0,06% Age at start of graduation; 13.3% Self-reported race (13.3% among medical graduates from public and 13.3% from private schools); 0.8% Type of institution attended for secondary education; 3.3% Medical school average ENADE 2023 score (8.5% among medical graduates from public schools and 0.9% among those from private schools)

Regarding educational characteristics of the medical schools attended, 68.6% of students in the cohort graduated from private medical schools, while 31.4% graduated from public institutions. Among students attending public medical schools, 28.1% were admitted through racial, disability, or income-based quotas, whereas among those graduating from private medical schools, 44.5% received some form of financial support during medical training.

As for geographic characteristics of training institutions, most students graduated from medical schools located in the Southeast and Northeast regions of the country.

Overall, entry into medical residency programmes varied mainly by admission pathway - being lower among quota-admitted students in public medical schools and higher among recipients of financial support in private institutions -, whereas other factors showed similar associations across public and private medical schoolgraduates.

55.7% of all students entered a specialty residency programme – 62.8% of the public medical school ones and 52.5% of the private ones. However, such proportions were lower for the public students admitted through quotas (52.8%), and higher for the private students in receipt of financial aid (55.0%).

Our logistic regression models identified similar factors predicting entrance in a residency programme for public and private medical school students (Table 2). Positive factors were being female, white, and having schooled in a private secondary school. Negative factors for both types of students were being over 21 years at graduation, being black, the medical school being in the Northern region, and in municipalities with fewer than 300,000 inhabitants, age of the medical school (< 21 years of running medical degrees), and lower ENADE scores attributed to the medical school.

**Table 2:** Adjusted logistic regression analysis for entry in medical residency and selection of family & community medicine, by public and private medical schools

Characteristics	MEDICAL PUBLIC SCHOOLS				MEDICAL PRIVATE SCHOOLS				
	Entry into medical residency aOR (95% CI)		Family Medicine & Community Medicine aOR (95% CI)		Entry into medical residency aOR (95% CI)		Family Medicine & Community Medicine aOR (95% CI)		
Sex		***		***		***		***	
Female	Ref.		Ref.		Ref.		Ref.		
Male	0.77 (0.73; 0.81)		0.81 (0.73; 0.89)		0.91 (0.88; 0.94)		0.67 (0.61; 0.73)		
Age at start of graduation		***		***		***		***	
≤ 21years	Ref.		Ref.		Ref.		Ref.		
> 21 years	0.46 (0.44; 0.49)		1.75 (1.57; 1.95)		0.39 (0.38; 0.40)		2.09 (1.92; 2.28)		
Self-reported race		***		**		***		***	
White	Ref.		Ref.		Ref.		Ref.		
Pardos (mixed-race)	0.79 (0.74; 0.84)		1.26 (1.12; 1.41)		0.84 (0.81; 0.88)		1.22 (1.09; 1.35)		
Black	0.80 (0.72; 0.89)		1.28 (1.05; 1.57)		0.84 (0.76; 0.94)		1.57 (1.23; 2.00)		
Yellow	0.69 (0.59; 0.81)		1.11 (0.81; 1.51)		0.98 (0.88; 1.09)		1.34 (1.05; 1.71)		
Indigenous	0.32 (0.24; 0.43)		1.51 (0.78; 2.95)		1.00 (0.76; 1.32)		2.04 (1.23; 3.38)		
Type of institution attended for secondary education		***		**		***			
Public school	Ref.		Ref.		Ref.		Ref.		
Private school	1.38 (1.28;1.49)		0.82 (0.71; 0.95)		1.11 (1.06; 1.16)		0.98 (0.88; 1.09)		
Region of the medical school				***		***		***	
Southeast	Ref.	***	Ref.		Ref.		Ref.		
North	0.58 (0.52; 0.65)		1.35 (1.10; 1.65)		0.88 (0.81; 0.94)		1.41 (1.16; 1.70)		
Northeast	0.69 (0.64; 0.74)		1.44 (1.26; 1.64)		0.99 (0.94; 1.04)		1.45 (1.29; 1.64)		
South	0.88 (0.81; 0.95)		1.24 (1.07; 1.43)		1.31 (1.25; 1.37)		1.34 (1.07; 1.35)		
Central-west	1.01 (0.92; 1.11)		1.47 (1.25; 1.73)		1.09 (1.02; 1.16)		1.55 (1.33; 1.80)		
Medical school location by population size		***				***		**	
Areas with > 300.000 inhabitants	Ref.		Ref.		Ref.		Ref.		
Areas with fewer than < 300.000 inhabitants	0.80 (0.74; 0.87)		1.03 (0.89; 1.19)		0.81 (0.79; 0.84)		0.86 (0.78; 0.95)		
Length of operation of the medical school		***		*		***			
≥ 21 years	Ref.		Ref.		Ref.		Ref.		
11 |-| 20 years	0.99 (0.90;1.08)		1.10 (0.93; 1.30)		0.82 (0.79; 0.85)		0.99 (0.89; 1.10)		
≤ 10 years	0.80 (0.73; 0.88)		0.80 (0.66; 0.96)		0.71 (0.68; 0.75)		1.10 (0.97; 1.24)		
Medical school average ENADE 2023 score		**		***		***			
Score 3–4-5 (higher)	Ref.		Ref.		Ref.		Ref.		
Score 1–2 (lower)	0.78 (0.67;0.92)		0.38 (0.24; 0.60)		0.67 (0.65; 0.70)		1.00 (0.90; 1.11)		
Admission through quotas		***		**					
No	Ref.		Ref.		---		---		
Yes	0.73 (0.67; 0.79)		1.28 (1.09; 1.49)		---		---		
Recipient of financial aid						***		*	
No	---		---		Ref.		Ref.		
Yes	---		---		1.23 (1.19; 1.27)		1.09 (1.00; 1.18)		
Number of observations (complete cases)	26,988		16,823		65,618		34,586		

Complete-case analyses were conducted. Observations (n) for each model are shown in the table and differ due to missing values in covariates

Statistical significance: *p < 0.05; **p < 0.01; ***p < 0.001

Our multivariate models showed that having been admitted in public medical schools through quotas was associated with lower odds of entering a medical residency programme compared with peers admitted through the regular path (adjusted Odds Ratio [aOR]: 0.73; 95% CI: 0.67; 0.79). On the other hand, being recipient of financial support in private medical schools was associated with higher odds of entering a medical residency programme, in comparison to the other students in private medical schools (aOR:1.23; 95% CI: 1.19; 1.27).

**Fig. 1:**
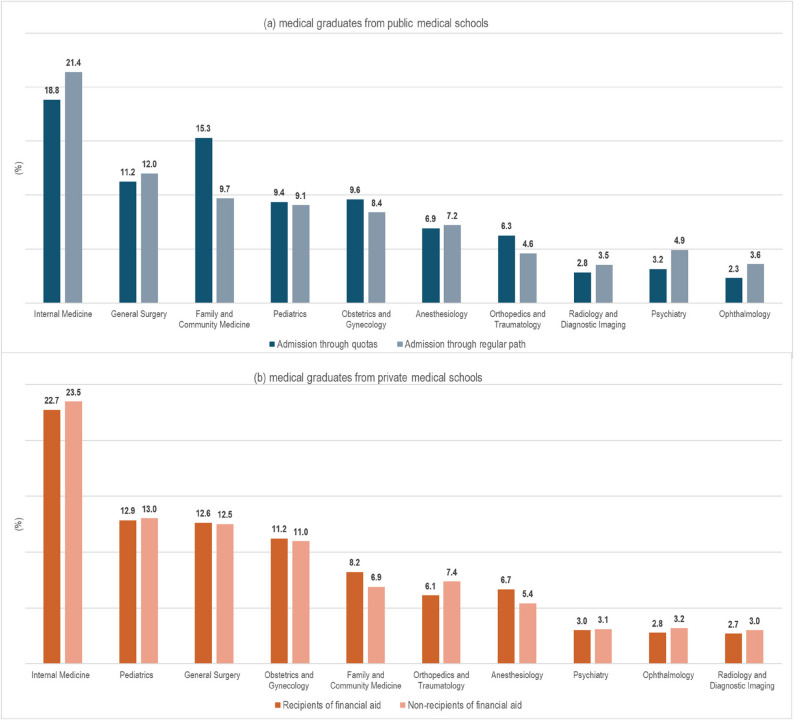
Proportion of students choosing the most common medical specialties, by type of medical school and admission

As for the specific specialties chosen by those students that entered a residency programme, the most common ones were similar for public and private medical school students, namely: Internal medicine, General surgery, Paediatrics, Family & Community Medicine, and Obstetrics & Gynaecology (see Fig. 1). Choice of specialty did not differ considerably between quota or financial support students and their peers in public and private medical schools, apart from Family & Community Medicine that was selected by 15.3% of quotas students (Vs 9.7% of their public-school peers) and by 8.2% of financial support students (Vs 6.9% of private school peers).

Multivariate logistic regression models for the selection of Family & Community Medicine show that being male is negatively associated with such specialty among public medical school students (aOR: 0.81; 95% CI: 0.73; 0.89) as well as private medical schools ones (aOR: 0.67; 95% CI: 0.61; 0.73). Coming from a private secondary school background is negatively associated with the selection of the primary care specialty among public (aOR: 0.82; 95% CI: 0.71; 0.95), but not for private medical school’s students (aOR: 0.98, 95% CI: 0.88; 1.09).

As for positive associations with entry in Family & Community Medicine, being over 21 years at graduation, and Black or Mixed-race were strong predictive factors for both public and private medical school students. Also having attended a medical school outside Brazil’s Southeast region was positively associated with the selection of Family & Community Medicine for both public and private medical school students.

Being a quota student in public medical schools was strongly associated with the selection of the primary care specialty (aOR: 1.28; 95% CI: 1.09; 1.49). Being recipient of financial subsidies in private universities was also a predictor of primary care choice (aOR: 1.09; 95% CI: 1.00; 1.18), although the magnitude of the effect was smaller.

## Discussion

Our analysis of Brazil’s Higher Education Census and National Medical Residency Commission databases showed that, between 2018 and 2022, 28.1% of all students in public medical schools were admitted through AA quotas, while 44.5% of students in private medical schools received financial support from the government. The rate of entry into the Medical Residency training programme was lower for quota students compared with their peers in public medical schools, whereas recipients of financial support had a higher entry rate than their peers in private medical schools. Female sex, white race, younger age at graduation, and attendance at private secondary schools were characteristics associated with entry into Medical Residency, along with higher quality scores attributed to the medical school.

The rate of selection of the primary care Family & Community Medicine specialty was higher among both quota and financial support students compared with their respective peers, although the difference was smaller for the latter group. Female sex, non-white race, older age, and attendance at a medical school outside the Southeast region were factors associated with the choice of the primary care specialty.

The necessity of starting to work before specialization because of financial hardship might in part explain the lower rate of entry in MR for quota students, typically less well-off than their peers in public medical schools [32]. Quota students may face greater financial pressures, which could lead them to start working immediately after graduation rather than pursuing residency right away. By the same token, being in receipt of financial support might have encouraged students in private medical schools to further their education. We acknowledge however that such cohort of students was very diverse, and included individuals from relatively wealthier backgrounds, possibly with access to better support to enter specialisation.

Both quotas and financial support policies were associated with higher selection of Family & Community Medicine, although the impact for the former was much larger than for the latter. The different profile of quotas and financial support students might have contributed to such preference, as these tend to come from rural and less wealthy backgrounds, characteristics typically associated with primary care specialties [27]. This profile may align with personal or community-oriented motivations, making Family & Community Medicine a more appealing choice for quota students.

Students’ academic ability and performance are likely to have been a factor in their decisions to further their medical education and choice of specialty [33]. The literature on performance of quota students in medical schools is inconclusive at best [34], with local studies providing evidence that quota students perform as well as their peers [35]. As we had only limited information on individual students’ performance, we can only speculate that those in public medical schools must have been of greater ability, as such schools received higher ENADE scores, and it is logical to think the best students would choose and have the ability to enter highly rated and almost fees-free public medical schools [26]. This would be consistent with our findings on the association between lower ENADE scores for (both public and private) medical schools and students diminished likelihood to enter medical residency. Contrary to the existing literature on this topic [36, 37], the link between student or school quality and the selection of primary care oriented specialties was not fully supported by our findings, though.

Despite their differences, both types of AA policies in Brazil were associated with higher likelihood of entering medical residency and the selection of primary care specialties. Governments around the world might therefore be presented with the option of choosing quotas in public universities if they want to increase numbers of primary care specialists and generalists, or financial support to students in private medical schools, if they want to provide students with an incentive to enter medical residency [34]. Ultimately, the main difference between the two is that quotas appeared to have increased diversity of medical students to a greater extent than scholarships to private students. And this remains the reason such measures were introduced in Brazil in the first place [38].

The introduction of race and income quotas in private universities as a requirement for the authorisation of new medical courses, or the increase of direct public subsidies to private medical schools, has been criticised as such measures may encourage the expansion of a lower-quality medical education market [39]. In turn, our findings appear to suggest that the provision of reserved places for quota students in public undergraduate medical courses would need to be accompanied by additional financial support to ensure both their entry into and retention within Medical Residency programmes. These outcomes are desirable for the health system because increasing the number of primary care physicians can help strengthen primary care capacity, potentially improving access and coverage, particularly in underserved regions.

We acknowledge a few limitations for this study. We included students graduating between 2018 and 2022, with residency data available up to 2024. For students graduating in later years (i.e., 2021–2022), follow-up was limited, so students who delayed their residency to after such dates may not have been captured, potentially underestimating enrolment rates. However, as our previous work [25] has showed that in Brazil just over half of physicians without prior residency enrolment entered a residency program within one year of graduation, and 22.1% began a residency within two years, we believe this risk of underestimation to be limited. Additionally, the early cohorts affected by the 2012 Quota Law were relatively small and institutional adjustments were ongoing, meaning that our logistic regression estimates may reflect early-stage effects and not fully capture the longer-term impact of affirmative action policies. Additionally, self-reported race was missing for 13% of students, which may have affected the precision of estimates involving race. Moreover, some students eligible for AA may have entered medical school through the regular admission route, potentially leading to misclassification and attenuation of estimated quota effects. Finally, due to the limited number of individual covariates, hierarchical/contextual models could not be applied, and a black-box adjustment approach was used; as such, residual confounding cannot be ruled out, which may limit the interpretation of the observed associations.

## Conclusions

Affirmative action measures have been used throughout the world to improve access to medical education for underrepresented groups and re-balance the medical workforce. This study provides a comprehensive analysis of the impact of affirmative action policies in Brazil on medical students’ entry into residency programs and their specialty choices. By examining the differences between students admitted through quota systems in public medical schools and those receiving financial aid in private institutions, we reveal significant disparities in residency enrolment rates and specialty selections.

Our findings indicate that students from underrepresented backgrounds who are admitted through quotas face lower odds of entering medical residency compared to their peers, while those receiving financial support in private schools exhibit higher chances of residency enrolment. Furthermore, both groups show a notable preference for Family & Community Medicine, highlighting the potential of affirmative action policies to influence the distribution of primary care specialists.

This research underscores the importance of these policies in promoting diversity within the medical workforce and suggests that, hom while quotas enhance student heterogeneity, financial support may serve as an effective incentive for students in private institutions to pursue further medical training. Ultimately, the insights gained from this study may inform policy decisions aimed at addressing the inequities in medical education and workforce distribution in Brazil and similar contexts globally.

## Data Availability

The datasets used and/or analysed during the current study are available from the corresponding author on reasonable request.

## Abbreviations

AA: Affirmative action
aOR: Adjusted Odds Ratio
CNRM/MEC: National Medical Residency Commission
e-mec: Brazil’s information database on medical courses
ENADE: National Exam of Students Performance
ENEM: National High School Exam
FIES: Students Financing Fund
IBGE: Brazilian Institute of Geography and Statistics
INEP: Higher Education Census
LMIC: Low- and Middle-Income Countries
MR: Medical Residency
PROUNI: Brazil’s University for All Scholarships Programme
SEDAP: Brazil’s Public Administration’s Protected Data Access Service
US: United States of America
